# LEFT OUT TO DRY: UNMET NEEDS AND RISK OF DEPRESSION AMONG OLDER ADULTS

**DOI:** 10.1093/geroni/igac059.2105

**Published:** 2022-12-20

**Authors:** Haowei Wang, Ashton Verdery, Rachel Margolis, Sarah Patterson

**Affiliations:** Tufts University, Quincy, Massachusetts, United States; The Pennsylvania State University, State College, Pennsylvania, United States; Western University, London, Ontario, Canada; University of Michigan, Ann Arbor, Michigan, United States

## Abstract

Pre-pandemic research has shown adverse consequences of having unmet care needs for older adults’ mental health. Due to the broad psychological distress and increased caregiving challenges during COVID-19, older adults’ vulnerabilities to unmet needs may be amplified by the pandemic, especially for those with functional limitations and intense care needs. This study aims to examine (1) the associations between unmet needs and depression among older adults before and during the COVID-19 and (2) whether the excess mental health consequences from unmet needs and COVID-19 vary by older adults’ dementia status. We pool data from the 2018, 2019, and 2020 rounds of National Health and Aging Trends Study, a nationally representative sample of U.S. Medicare beneficiaries. We analyze N=6,273 older adults aged 70 years and older who had limitations with self-care, household activities, or mobility. Results show that older adults with functional limitations experienced increased risk of depression over time. Before and during the pandemic, older adults with unmet needs and older adults with probable dementia had higher risks of depression compared to their counterparts, respectively. The risk of depression was highest among older adults who had probable dementia and could not have their care needs met. For older adults without dementia, their risks of depression increased significantly from pre-pandemic to COVID-19 if they had unmet care needs. Findings demonstrate the disproportionate impacts of COVID-19 on mental health among older adults. Older adults who have cognitive impairments and unmet needs are in particular need of mental health support.

